# Supplementary material for: Evidence supported by Mendelian randomization: impact on inflammatory factors in knee osteoarthritis
Source: Front Med (Lausanne). 2024 May 28;11:1382836. doi: 10.3389/fmed.2024.1382836 (PMC11165061; doi:10.3389/fmed.2024.1382836)
Supplement: Supplementary file 1 [file Data_Sheet_1.ZIP › data/GCST90274805_5e6/805.leaveoneout.pdf]

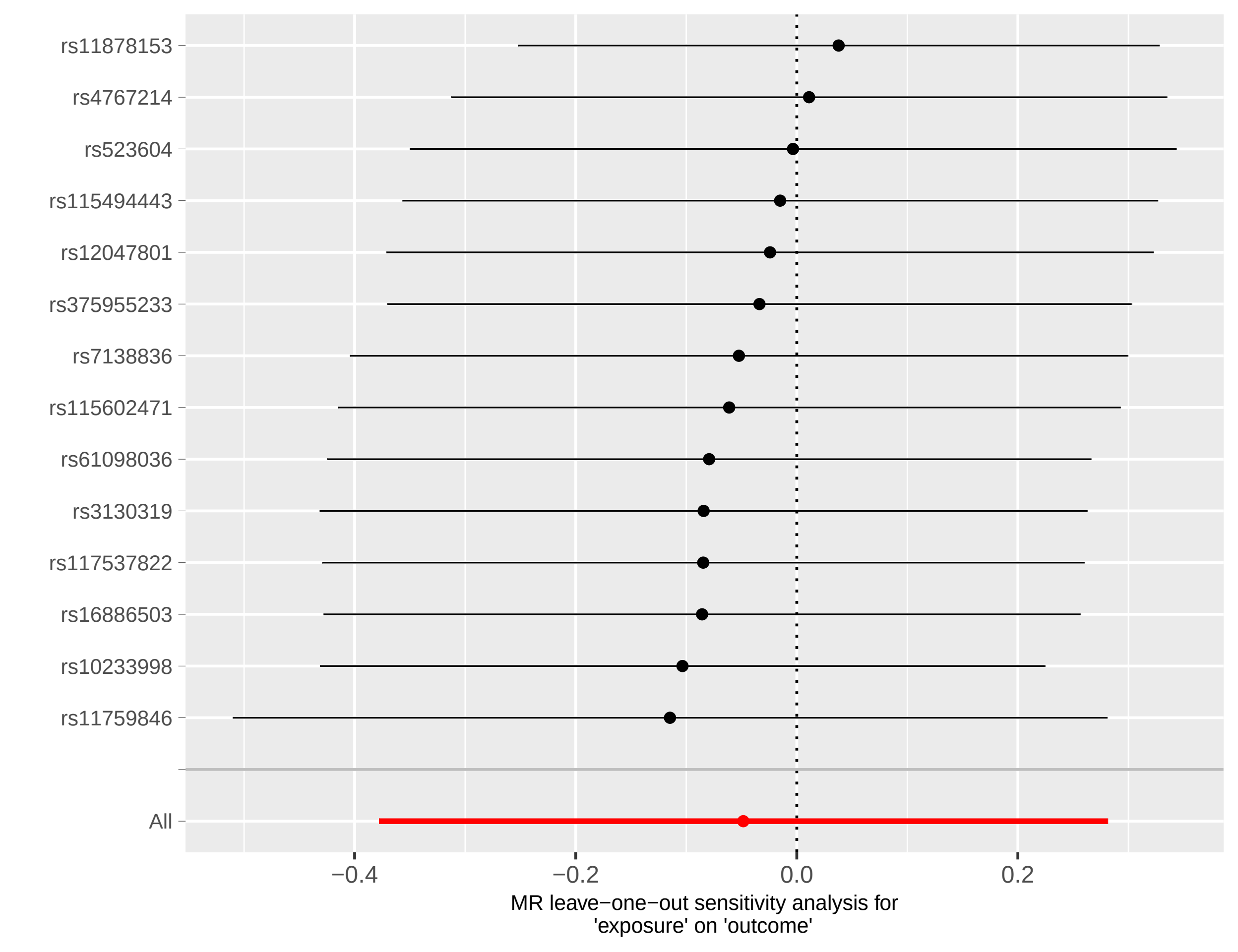
rs11878153
rs4767214
rs523604
rs115494443
rs12047801
rs375955233
rs7138836
rs115602471
rs61098036
rs3130319
rs117537822
rs16886503
rs10233998
rs11759846
All
−0.4
−0.2
0.0
0.2
MR leave−one−out sensitivity analysis for
'exposure' on 'outcome'
